# Development of a Nomogram for Predicting Asymptomatic Coronary Artery Disease in Patients with Ischemic Stroke

**DOI:** 10.2174/1574887117666220513104303

**Published:** 2022-11-25

**Authors:** Jie Yang, Xinguang Yang, Jun Wen, Jiayi Huang, Lihong Jiang, Sha Liao, Chun Lian, Haiyan Yao, Li Huang, Youming Long

**Affiliations:** 1 Department of Neurology, The Second Affiliated Hospital of GuangZhou Medical University, 250# Changgang East Road, GuangZhou, 510260, Guangdong Province, China;; 2 Key Laboratory of Neurogenetics and Channelopathies of Guangdong Province and The Ministry of Education of China, Institute of Neuroscience and the Second Affiliated Hospital of GuangZhou Medical University, 250# Changgang east Road, GuangZhou, 510260, Guangdong Province, China;; 3 Department of Neurology, Jiangmen Central Hospital, 23# Haibang Street, North Street, Jiangmen, 529000, Guangdong Province, China;; 4 Department of Neurology, Dongguan Dongcheng Hospital, 56# Nancheng Road, DongGuan, 523000, Guangdong Province, China

**Keywords:** Acute ischemic stroke, coronary artery disease, coronary computed tomography angiography, cerebral angiography, nomogram

## Abstract

**Background:**

Coronary artery stenosis (CAS) ≥50% often coexists in patients with ischemic stroke, which leads to a significant increase in the occurrence of major vascular events after stroke. This study aimed to develop a nomogram for diagnosing the presence of ≥50% asymptomatic CAS in patients with ischemic stroke.

**Methods:**

A primary cohort was established that included 275 non-cardioembolic ischemic stroke patients who were admitted from January 2011 to April 2013 to a teaching hospital in southern China. The preoperative data were used to construct two models by the best subset regression and the forward stepwise regression methods, and a nomogram between these models was established. The assessment of the nomogram was carried out by discrimination and calibration in an internal cohort.

**Results:**

Out of the two models, model 1 contained eight clinical-related variables and exhibited the lowest Akaike Information Criterion value (322.26) and highest concordance index 0.716 (95% CI, 0.654-0.778). The nomogram showed good calibration and significant clinical benefit according to calibration curves and the decision curve analysis.

**Conclusion:**

The nomogram, composed of age, sex, NIHSS score on admission, hypertension history, fast glucose level, HDL cholesterol level, LDL cholesterol level, and presence of ≥50% cervicocephalic artery stenosis, can be used for prediction of ≥50% asymptomatic coronary artery disease (CAD). Further studies are needed to validate the effectiveness of this nomogram in other populations.

## 1 INTRODUCTION

Ischemic heart disease and stroke are the leading causes of mortality among adults aged 50 and over around the world [1] Patients suffering a transient ischemic attack (TIA) or ischemic stroke were found to be at high risk of coronary artery disease (CAD) [2] CAD is strongly associated with a high risk of long-term myocardial infarction (MI) after ischemic stroke or TIA, [3, 4] long-term recurrent ischemic stroke, [5] and is the leading cause of long-term mortality in patients with stroke [6]. Compared to patients with ischemic stroke with no coronary artery stenosis (CAS), the risk for 2-year combined major vascular events was 4.36× higher for those with asymptomatic CAS ≥50% [7]. Invasive coronary angiography cannot be routinely performed in patients with cerebral artery atherosclerosis [8]. Therefore, several studies have begun to explore predictive models to screen patients with ischemic stroke who are at high risk of presence of ≥50% asymptomatic CAD [2, 9-11]

The traditional statistical strategies only adopted the variables which were significant on univariate analysis to establish the final prediction models, which led to model overfitting, thus showing poor results [12]. Several advanced statistical methodologies have been developed to minimize this limitation, such as the best subsets regression (BSR) and the forward stepwise regression (FSR) methods [13]. The nomogram is a graphical statistical instrument that incorporates variables to develop a continuous scoring system and calculates the precise risk probability of a particular outcome for an individual patient [14]. This instrument is an important component of modern medical decision making and has been used in an extensive array of applications including cancer, stroke, surgery, and other specialties [13, 15-17]. To date, a nomogram model with adequate power to detect the probability of asymptomatic CAD in ischemic stroke patients has not yet been designed.

Therefore, our study aimed to develop an effective nomogram for prediction of asymptomatic CAD in non-cardioembolic stroke patients by using preexisting vascular risk factors, feasible baseline measurements, and adopting advanced statistical analysis.

## 2 PATIENTS AND METHODS

### 2.1 Patient Selection

This was a single-center, cross-sectional study. Data from patients admitted to the stroke unit of the second affiliated hospital of Guangzhou Medical University from January 2011 to April 2013 were collected for this study if they meet the following selection criteria: at least 18 years old; experienced an ischemic stroke within 14 days after the onset of symptoms; no prior history of CAD (*i.e*., angiographically confirmed CAD, unstable angina, coronary artery stent, or angioplasty/coronary artery bypass graft); suspected to have non-atherosclerotic arterial stenosis, such as arterial dissection and vasculitis, patients who undergone revascularization procedures, or undergone radiotherapy for nasopharyngeal cancer, patients without both cervicocephalic digital subtract angiography (DSA) and coronary multi-detector computed tomography angiography (MDCTA), were not eligible. The study was approved by the ethics committee of the Second Affiliated Hospital of Guangzhou Medical University (No. 2021-hs-27) and was conducted in accordance with the ethical guidelines of the 1975 Declaration of Helsinki. The informed consent of patients was waived because of its retrospective design.

### 2.2 Demographics and Clinical Characteristics

Demographic data and data of vascular risk factors (hypertension history, diabetes mellitus history, hyperlipidemia, ischemic stroke history, CAD, active or past smoking) were collected from medical records. Routine blood tests, fast blood glucose, and lipid profile were measured within 48 h after admission. Systolic and diastolic blood pressures were measured in the supine position upon admission. All patients were subjected to brain magnetic resonance imaging with spin-echo diffusion-weighted imaging or computed tomography scan and 12-lead electrocardiography.

### 2.3 Cervicocephalic Atherosclerosis Assessment

Imaging of cervical and intracranial arteries consisted of cervical and intracranial DSA in all patients. Cervicocephalic atherosclerosis was assessed using standardized methods [9]. The cervicocephalic arteries were divided into nine segments: common carotid, extracranial carotid, intracranial carotid, middle cerebral, anterior cerebral, extracranial vertebral, intracranial vertebral, basilar, and posterior vertebral arteries. The existence of cervicocephalic artery disease (CVD) was confirmed when there was stenosis ≥50% in any of the abovementioned arteries [9].

### 2.4 Coronary Stenosis Measurement

The presence of coronary artery atherosclerosis was assessed using 64-section CT coronary angiography. All 64-section CT coronary angiographies were reviewed by two experienced radiologists blinded to the clinical data and results of cervicocephalic atherosclerosis assessment. The existence of CAS was confirmed when there was stenosis ≥50% in at least one segment at the four main coronary branches, which are the left main, left anterior descending, left circumflex, and right coronary arteries [18].

### 2.5 Selection of Variables

To construct the nomogram, we used two methods to select the significant predictors of asymptomatic CAD. In order to avoid over-fitting or under-fitting of the model, three advanced statistical methods, best subset regression (BSR) and forward stepwise regression, were adopted to select variables in the primary cohort. The criteria of variable selection for the BSR and the forward stepwise regressions were determined by the Bayesian information criterion (BIC) [13].

### 2.6 Model Development

CAD-related predictive models were established in the primary cohort based on the selected variables by adopting binary logistic regression. Eventually, we developed two models: (a) model 1 consisted of eight variables according to the BSR, (b) model 2 consisted of six variables according to the forward stepwise regression. The final model was determined by the Akaike Information Criterion (AIC), [19] the receiver operating characteristic (ROC) curves, and the Harrell concordance index (C-index). The nomogram was derived from the final model.

### 2.7 Performance of the Nomogram

The model was internally validated using all data of the training cohort by 10-fold cross-validation. Discriminative performance was measured by C-index. Calibration was tested using a calibration plot with bootstraps of 500 resamples, which described the degree of fit between actual and nomogram-predicted CAD.

### 2.8 Clinical Usage

The decision curve analysis (DCA) was used to assess the clinical usage of nomogram. Detailed descriptions of the DCA have been previously reported [15]. Results were considered statistically significant at *p* < 0.05.

### 2.9 Statistical Analysis

Categorical variables were expressed as numbers (percentage) and continuous variables as medians (quartile). Differences in baseline characteristics between groups for continuous variables were assessed using Mann-Whitney U test, and the Chi-squared test or Fisher’s exact test was used for categorical variables according to their sample size. Proportions were calculated for categorical variables, dividing the number of events by the total number after excluding missing/unknown cases.

Differences between groups were considered statistically significant at *p* < 0.05. SPSS 21.0 (IBM Corp., Armonk, NY, USA), R statistical software (http://www.R-project.org, The R Foundation), and Free Statistics software version 1.4 was used for statistical analysis.

## 3 RESULTS

### 3.1 Clinical Characteristics

The flow chart outlining the patient inclusion process is shown in Fig. (**1**). A total of 275 patients (200 males and 75 females) were included in the study. The mean age of enrolled subjects was 63.3±10.1 years. A total of 88 acute stroke patients had manifestations of CAS, giving a prevalence rate of 32.0%.

The risk factors for CAD and the comparisons between groups with and without CAD are shown in Table **1**. Age, hypertension history, diabetes mellitus history, National Institutes of Health Stroke Scale Score (NIHSS) on admission, fast blood glucose level, high-density lipoprotein cholesterol (HDL-C) level, and presence of ≥50% stenosis in the cervicocephalic artery were significantly enriched in the group with CAD compared to the groups without CAD (all *p* < 0.05). All risk factors shown in Table **1** were adopted in the variables selection for prediction model development.

### 3.2 Selection of Variables Using the BSR and the Stepwise Regression

**Fig. (2):**
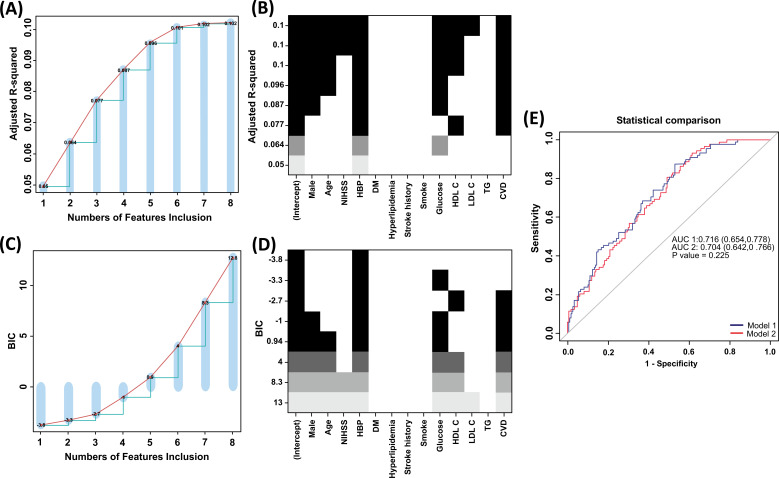
Variable selection methods. (A and B) The selection of variables using the BSR method (Adjusted R^2^). (C and D) The selection of variables using the BSR method (BIC). (**E**) The comparison between ROC curves of CAD in primary cohort. BIC, Bayesian information criterion; BSR, best subsets regression; NIHSS, National Institutes of Health Stroke Scale; HBP, hypertension; DM, Diabetes mellitus; LDL-C, low density lipoprotein cholesterol; HDL-C, highdensity lipoprotein cholesterol; CVD, cervicocephalic artery disease.

The BSR method showed great benefits in variable selection because all possible combinations of variables were calculated and the final selected combination was considered optimal based on the max adjusted R^2^ (Fig. **2A** and **B**) or minimum BIC (Fig. **2C** and **D**). As shown in Fig. (**2A** and **B**), the adjusted R^2^ method selected eight parameters, including male, age, hypertension, NIHSS at admission, fast blood glucose level, HDL-C level, low-density lipoprotein cholesterol (LDL-C) level, and CVD. However, only one parameter was selected by minimum BIC: hypertension history. We also used the stepwise regression to choose combinations of six potential predictors, including sex, age, hypertension, fast blood glucose level, HDL level, and CVD (Supplementary Table **1**). Because it is inappropriate to use a single variable to develop the prediction model, we excluded the result from minimum BIC for further analysis. The choice of final prediction model was determined by the ROC curve and the C-index, which were also used to examine the efficiency of the two models, with model 1 based on adjusted R^2^ and model 2 based on stepwise regression (Fig. **2E**).

**Fig. (3):**
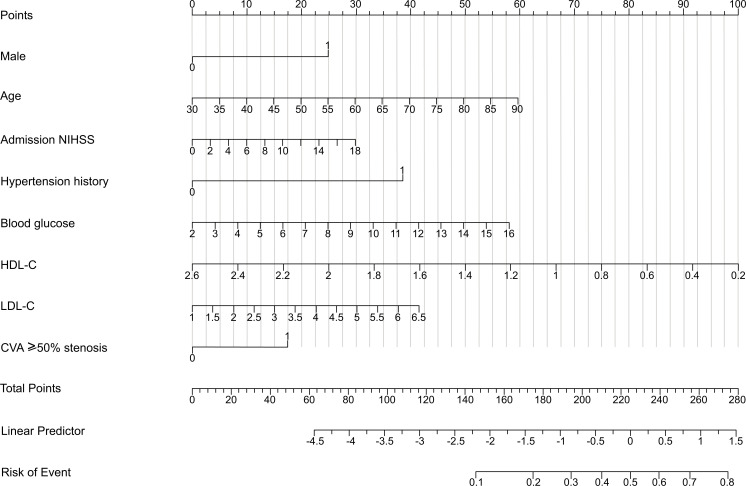
CAD-related nomogram prediction score. CAD-related nomogram was constructed to predict asymptomatic CAD for stroke patients, with the male, age, NIHSS on admission, hypertension history, blood glucose level, HDL-C level, LDL-C level and CVA≥50% stenosis. The “total points” are calculated as the sum of the individual score of each of the 8 variables included in the nomogram. NIHSS, National Institutes of Health Stroke Scale; HDL-C, highdensity lipoprotein cholesterol; LDL-C, low lipoprotein cholesterol; CVA, cervicocephalic artery.

The nomogram obtained from the final model was optimal (Fig. **3**). Higher total points based on the sum of the assigned number of points for each predictor in the nomogram were associated with an increased risk of mortality. For example, a male patient aged 70 years with a baseline NIHSS score of 14, hypertension history, fast blood glucose level 8.0mmol/L, HDL-C level 1.0mmol/L, LDL-C level 4.0mmol/L and presence of CVA ≥50% stenosis would have a total of 258 points (25 points for male sex, 40 points for age, 23 points for baseline NIHSS, 38 points for hypertension history, 25 points for blood glucose level, 67points for HDL-C level, 22.5 points for LDL-C level, and 17.5 points for presence of CVA ≥50% stenosis). The predicted CAD is 70.0% for this patient.

### 3.3 Performance of Nomogram

Prediction potential of the nomogram was measured by calculating the C-index, which was 0.716 (95% CI, 0.654-0.778), indicating relatively good predictive power. Fig. (**4a**) shows a calibration plot, which compares the nomogram prediction and actual observation of CAD. The calibration plot revealed good predictive accuracy of the nomogram.

DCA can estimate the net benefit of a model based on the difference between the number of true- and false-positive results and is widely used in assessing whether the nomogram-assisted decision would improve patient outcomes. As shown in Fig. (**4b**), the DCA indicated that when the threshold probabilities ranged between 2.1% and 78.0% in the training cohort, the use of the nomogram to predict CAD provided a greater net benefit than the “treat all” or “treat none” strategies, which indicates the clinical usefulness of the nomogram.

## 4 DISCUSSION

In this study, we developed a precise nomogram, based on age, sex, NIHSS score at admission, hypertension history, blood glucose level, HDL cholesterol level, LDL cholesterol level, and presence of stenosis ≥50% in the cervicocephalic artery to predict the probability of asymptomatic CAD in patients with acute ischemic stroke. The calibration of the nomogram and its ability to predict CAD were demonstrated in the developing cohort. Given that all parameters were evaluated within 48 h of admission, this nomogram could be applied in the early stage of stroke for predicting asymptomatic CAD.

Our study shows the incidence of asymptomatic CAD was 32.0%, which is within the range reported by recent studies (18.0% to 52%) [9-11, 18]. Current models for predicting asymptomatic CAD in stroke have limitations. Both PRECORIS study [9] and the study by Choi and colleagues [18] developed models based on cut-off values of discrete variables, such as age, sex, and LDL cholesterol level. However, this may reduce predictive accuracy, given that they do not fully utilize within-category information. By converting the total score into a continuum of individual scores through a logarithmic formula, we developed a relatively precise nomogram (C-index, 0.716) for predicting probability from 2.1% to 78.0% of asymptomatic CAD in acute ischemic stroke patients. Patients with CAD can be referred early to a cardiologist for further diagnosis and treatment, which may reduce the incidence of serious coronary events in short-term [20] and long-term [3].

Advanced age, male sex, and hypertension history have previously been reported to be associated with the incidence of asymptomatic CAD in acute ischemic stroke patients [9]. Consistent with previous reports, age, sex, and hypertension history in our nomogram were significant predictors of asymptomatic CAD. However, NIHSS was also a significant predictor of asymptomatic CAD in our study but has seldomly been reported in previous similar studies [9-11, 18]. Because higher NIHSS is strongly associated with large artery disease and poor outcome of ischemic stroke, [20] and the coronary and cervicocephalic arteries are often simultaneously affected by similar vascular risk factors, [22] it is necessary to consider NIHSS as a predictor of CAD in ischemic stroke patients.

In addition to nonmodifiable variables (age, sex, high blood pressure, and baseline NIHSS score) of the individual patient, the levels of fasted glucose, HDL-C, and LDL-C upon admission were also predictors in the nomogram. Hyperglycemia shares many common mechanisms with other atherogenic factors, such as endothelial activation and inflammation, mitochondrial oxidative stress, changes in extracellular matrix components, and disruption of cellular defense systems [23]. In diabetic patients, each 1-standard deviation increase in fasting plasma glucose conveyed 2.11-fold higher risks of significant coronary stenosis after adjustment for other conventional cardiovascular risk factors [24]. In the prediabetic state, the severity of angiographic CAD also increased along with the increasing fast plasma glucose levels [25]. Studies of LDL-C in stroke patients with asymptomatic coronary heart disease are limited and results are inconsistent [9, 11]. One study has shown the association between higher LDL-C and stroke combined with asymptomatic CAS, [9] but another study has shown a non-significant correlation [11]. Our study shows that high LDL-C is significantly associated with asymptomatic CAD and is independent of blood glucose level and HDL-C level. A prospective community-based cohort study showed an interaction between triglyceride (TG), HDL-C, and LDL-C levels in the occurrence of CAD. High TG and low HDL-C levels were associated with risk of CAD, which was a phenomenon seen in people with high LDL-C levels but not observed in those with lower LDL-C levels [26]. Recent meta-analysis showed that a high HDL-C level is associated with reduced risk of total stroke and total ischemic stroke [27]. Another prospective community-based study has shown that HDL-C is not associated with a reduction in the occurrence of stroke with large vessel subtype in cerebral infarction, but a reduction in the occurrence of lacunar infarct subtype in cerebral infarction, suggesting that the mechanism of HDL-C in reducing the occurrence of cerebral infarction is not related to its protective function against lipid-rich atherosclerosis [28].

The presence of ≥50% cervicocephalic artery stenosis has been proved to be strongly associated with asymptomatic CAD in nondisabled stroke patients [2, 9]. In our study, we found that the presence of ≥50% cervicocephalic artery stenosis is related to the presence of asymptomatic CAD in all noncardioembolic ischemic stroke patients, which extends the use of this result in a broader stroke population. Some characteristics, such as smoking habit and previous incidence of stroke, were not found to be associated with CAD, which is probably attributable to the differences in sample size, study population, and study methods. A recent study suggests that patients with TIA/stroke with cardiovascular disease are at high risk of recurrent ischemic events despite the use of antithrombotic and lipid-lowering therapy. Thus, intensive lipid-lowering therapy may be justified. However, increased antithrombotic therapy will increase the risk of extracranial hemorrhage, thereby offsetting its benefits [5].

Several limitations should be addressed when interpreting the results of this study. First, we only included patients of Asian ethnicity, which may limit the generalizability of the results. Second, this is a retrospective study. The findings of our study need to be corroborated by prospective studies and randomized controlled trials. Third, large-scale and multi-center clinical trials are needed to be performed to validate and modify the model. Despite these limitations, our analysis has a few strengths. We developed a novel and relatively accurate nomogram. In addition, the nomogram was based on variables that can be easily abstracted and used in a real-world setting.

## CONCLUSION

In conclusion, approximately one-third of noncardioembolic ischemic stroke patients have ≥50% asymptomatic CAD. In addition, our study developed a nomogram, composed of age, sex, NIHSS score on admission, hypertension history, fast blood glucose level, HDL cholesterol level, LDL cholesterol level, and presence of ≥50% cervicocephalic artery stenosis, which could be used for prediction of ≥50% asymptomatic CAD. Further studies are warranted to validate the effectiveness of this nomogram in other populations.

## Figures and Tables

**Fig. (1):**
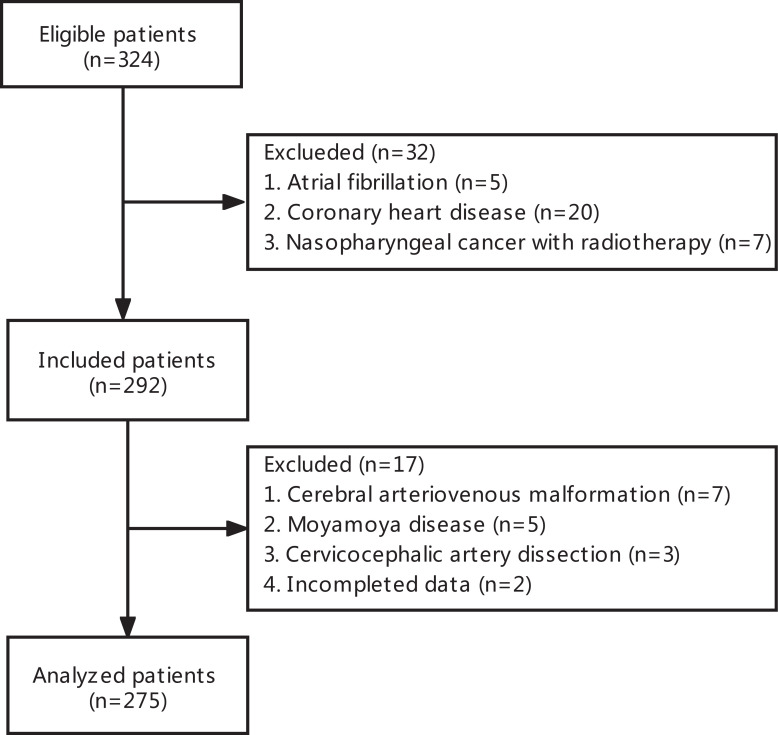
Flowchart of patients’ enrollment.

**Fig. (4):**
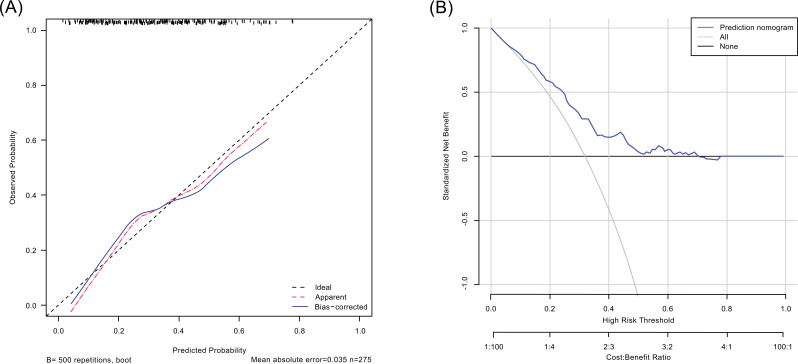
Calibration plot (**A**) and decision curve analysis (**B**) of the nomogram. (**A**) The dotted line represents the performance of the nomogram, whereas the solid line corrects for any bias in the nomogram. The dashed line represents the reference line where an ideal nomogram would lie. (**B**) The x-axis indicates the threshold probability. The y-axis measures the net benefit. The gray line displays the net benefit of the strategy of treating all patients. The black line illustrates the net benefit of the strategy of treating no patients. The blue line indicates the nomogram. Decision curve analysis is a specific method developed for evaluating the prognostic value of nomogram strategies. This nomogram was developed to assess the probability of the asymptomatic CAD of a given patient. A stroke patient with a high risk of asymptomatic CAD may need“further treatment,” such as intensified lipid-lowering therapy or percutaneous coronary intervention; a patient with a low risk of asymptomatic CAD may not need“further treatment.” Distinguishing patients with a high and low risk of asymptomatic CAD is the main purpose of this nomogram. In the present study, the reference risk was calculated by assuming that all patients need further treatment for preventing CAD, whereas zero net benefit was defined as no patients needing further therapy. The threshold probability is when the expected benefit of further therapy is equal to the expected benefit of avoiding further therapy. For any given probability threshold, the nomogram with the greatest net benefit would be the most preferred model. CAD indicates coronary artery disease.

**Table 1:** Risk factors for ≥50% asymptomatic coronary artery disease (Univariate Analysis).

**Variables**	**Total** **(n = 275)**	**Non-CAD** **(n = 187)**	**CAD** **(n = 88)**	***p*-value**
Male, n (%)	200 (72.7)	132 (70.2)	71 (78)	0.310
Age, mean (SD)	63.3 (10.1)	62.4 (10.2)	65.2 (9.5)	0.030
Ischemic stroke history, n (%)	55 (20.0)	32 (17.1)	23 (26.1)	0.113
Hypertension, n (%)	207 (75.3)	128 (68.4)	79 (89.8)	< 0.001
Diabetes mellitus, n (%)	85 (30.9)	48 (25.7)	37 (42.0)	0.009
Hyperlipidemia, n (%)	142 (51.6)	94 (50.3)	48 (54.5)	0.594
Smoking (active or past), n (%)	93 (33.8)	61 (32.6)	32 (36.4)	0.634
NIHSS on admission (median, IQR)	3.0 (1.0, 5.0)	3.0 (1.0, 5.0)	4.0 (2.0, 7.0)	0.013
Glucose, mean (SD)	5.9 (2.4)	5.6 (2.1)	6.5 (2.8)	0.006
Triglycerides, median (IQR)	1.4 (1.0, 1.8)	1.3 (1.0, 1.8)	1.4 (1.2, 1.9)	0.102
HDL-C, mean (SD)	1.0 (0.3)	1.0 (0.3)	0.9 (0.2)	0.007
LDL-C, mean (SD)	2.8 (0.9)	2.8 (0.8)	2.9 (1.0)	0.238
Presence of CVA ≥50% stenosis, n (%)	144 (52.4)	87 (46.5)	57 (64.8)	0.007

**Note:** CAD, coronary artery disease; NIHSS, National Institutes of Health Stroke Scale; LDL-C, low-density lipoprotein cholesterol; HDL-C, high-density lipoprotein cholesterol; CVA, cervicocephalic artery.

## Data Availability

The dataset that support the results and findings of this research are available from the corresponding author [YL], upon reasonable request.

## LIST OF ABBREVIATIONS

CAD: coronary artery disease
CAS: coronary artery stenosis
TIA: transient ischemic attack
