# Supplementary material for: Development of a Nomogram for Predicting Asymptomatic Coronary Artery Disease in Patients with Ischemic Stroke
Source: Curr Neurovasc Res. 2022 Nov 25;19(2):188–95. doi: 10.2174/1574887117666220513104303 (PMC9900699; doi:10.2174/1574887117666220513104303)
Supplement: Supplementary file 1 [file CNR-19-188_SD1.pdf]

Supplementary Material

Development of a Nomogram for Predicting Asymptomatic Coronary Artery Disease in Patients with Ischemic Stroke

Jie Yang[1,2], Xinguang Yang[1,2], Jun Wen[3], Jiayi Huang[1,2,4], Lihong Jiang[1,2], Sha Liao[1,2], Chun Lian[1,2], Haiyan Yao[1,2], Li Huang[1,2], Youming Long[1,2,*].

[1]Department of Neurology, The Second Affiliated Hospital of GuangZhou Medical University, 250# Changgang East Road, GuangZhou, 510260, Guangdong Province, China; [2]Key Laboratory of Neurogenetics and Channelopathies of Guangdong Province and The Ministry of Education of China, Institute of Neuroscience and the Second Affiliated Hospital of GuangZhou Medical University, 250# Changgang east Road, GuangZhou, 510260, Guangdong Province, China; [3]Department of Neurology, Jiangmen Central Hospital, 23# Haibang street, North Street, Jiangmen, 529000, Guangdong Province, China; [4]Department of Neurology, Dongguan Dongcheng Hospital, 56# Nancheng Road, DongGuan, 523000, Guangdong Province, China

Supplementary Table 1

Stepwise regression model for variables selection.

Variable	Estimate	Std Error	z-value	*p*-value	AIC
(Intercept)	-3.91	1.378	-2.837	0.005	322.2
Male	0.682	0.366	1.861	0.063	
Age	0.029	0.015	1.885	0.059	
Hypertension	1.07	0.404	2.647	0.008	
Blood glucose level	0.134	0.059	2.251	0.024	
HDL-C	-1.146	0.653	-1.756	0.079	
Presence of CVA ≥50% stenosis	0.513	0.286	1.795	0.073	

HDL-C, high-density lipoprotein cholesterol; CVA, cervicocephalic artery; AIC, Akaike Information Criterion.

1875-5739/22 **© 2022 Bentham Science Publishers**
